# Pulmonary Cavitary Disease Secondary to Mycobacterium xenopi Complicated by Respiratory Failure

**DOI:** 10.7759/cureus.3512

**Published:** 2018-10-29

**Authors:** Saad Habib, Kartikeya Rajdev, Sami Pervaiz, Abdul Hasan Siddiqui, Mohammed Azam, Michel Chalhoub

**Affiliations:** 1 Internal Medicine, Staten Island University Hospital, Northwell Health, Staten Island, USA

**Keywords:** mycobacterium xenopi, non-tuberculous mycobacteria, copd, respiratory failure, pulmonary cavitary disease

## Abstract

Non-tuberculous mycobacteria (NTM) are a significant cause of pulmonary infection worldwide and can be clinically challenging. Mycobacterium xenopi (M. xenopi) has low pathogenicity and usually requires either host immune impairment, as in the case of a human immunodeficiency virus infection, or a structural lung disease to cause a clinical disease. Comorbidities have an essential role in M. xenopi occurrence. Herein, we present a rare case of pulmonary cavitary disease caused by M. xenopi complicated by respiratory failure and superinfection in a patient with a chronic obstructive pulmonary disease.

An 81-year-old woman presented to the hospital with the chief concerns of shortness of breath and productive cough lasting a few weeks before presentation. A computed tomography scan of the chest showed a right upper lobe, thick-walled, cavitary lesion measuring 2.1 cm x 4.3 cm x 3.1 cm with associated bronchiectasis and pleural parenchymal scarring. One year ago, the patient underwent bronchoscopy for a right upper lobe cavitary lesion, which revealed M. xenopi on bronchoalveolar lavage culture. During the current admission, she was started on rifampin, isoniazid, ethambutol, and clarithromycin because the M. xenopi was clinically significant and fulfilled the American Thoracic Society diagnostic criteria for NTM lung disease.

A diagnosis of NTM pulmonary disease does not necessarily suggest that treatment is required. The distinction between colonization and illness may be difficult upon the isolation of M. xenopi. A patient-centered approach is essential given that M. xenopi is often considered a commensal pathogen. When treatment is required, a multidrug approach with an individualized, optimal duration of therapy should be considered.

## Introduction

Non-tuberculous mycobacteria (NTM) are emerging worldwide as significant causes of chronic pulmonary infection, posing several challenges. NTM displays a sizeable geographic variation in prevalence. Mycobacterium avium complex (MAC) is the most common type of NTM pathogen causing lung disease worldwide, followed by Mycobacterium kansasii [1-2]. In some parts of Canada and Europe,Mycobacterium xenopi (M. xenopi) is the most frequent cause of lung disease second to MAC but is infrequently seen in the USA [1,3-4]. M. xenopi is known to be a commensal pathogen, has low pathogenicity, and usually requires either host immune impairment (as seen in a human immunodeficiency virus (HIV) infection) or a structural lung disease [3] to cause a clinical disease. Comorbidities have an essential role in an M. xenopi infection. Herein, we present a rare case of pulmonary cavitary disease caused by M. xenopi in a patient with chronic lung disease, which was complicated by sepsis, acute hypoxic respiratory failure, and overlapping pneumonia.

## Case presentation

**Figure 1:**
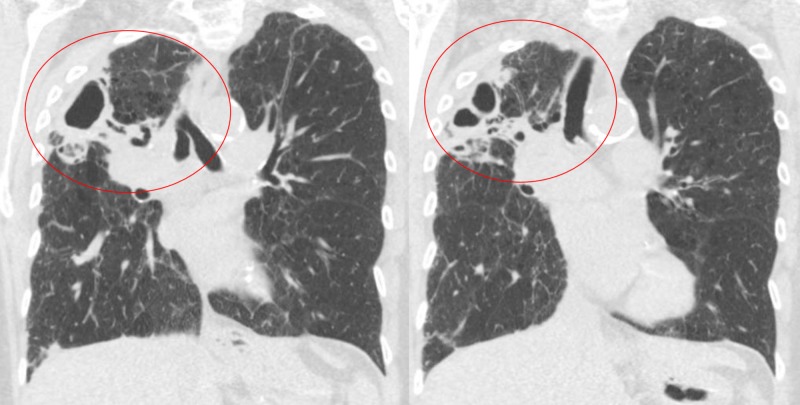
Initial coronal computed tomography of the chest showing right upper lobe cavitary lesion (circled)

**Figure 2:**
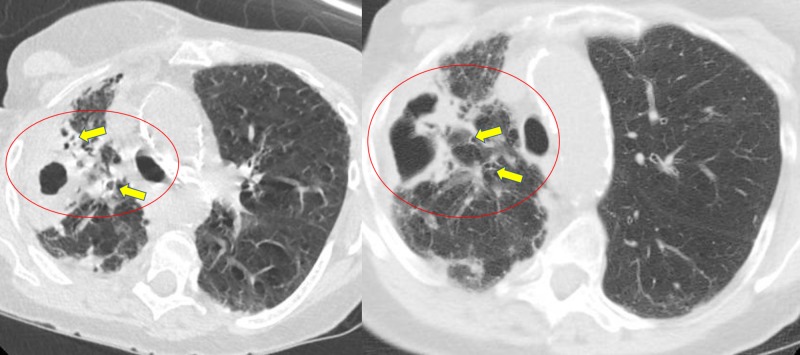
Initial axial computed tomography of chest showing right upper lobe cavitary opacity (circled) with adjacent bronchiectasis (yellow arrows)

An 81-year-old Caucasian woman presented to the hospital with chief concerns of shortness of breath and productive cough associated with generalized weakness, fatigue, and decreased appetite for a few weeks before presentation. Her medical history was significant for smoking one pack per day for 50 years and chronic obstructive pulmonary disease on two liters of home oxygen via nasal cannula. On physical examination, the patient was afebrile, tachypneic, tachycardic, had bilateral lung crackles more pronounced on the right, and was hypoxic at 86% on two liters of supplemental oxygen. Laboratory testing revealed an elevated leukocyte count of 15.82 k/µL with 91% neutrophils. Serum electrolytes, kidney function, and liver function tests were within reference ranges. The initial chest x-ray showed a right upper lobe opacity with pleural thickening, scarring, right lung volume loss, and bronchiectasis. The patient was started on azithromycin and cefepime for community-acquired pneumonia and sepsis, she was started on non-invasive ventilation via bilevel positive airway pressure for acute hypoxic respiratory failure and admitted to the medical floor. Further imaging with computed tomography (CT) scan of the chest showed a right upper-lobe thick-walled cavitary lesion measuring 2.1 cm x 4.3 cm x 3.1 cm with associated bronchiectasis and pleural parenchymal scarring (Figure 1, Figure 2).

One year ago, the patient underwent bronchoscopy for right upper lobe cavitary lesion that incidentally revealed M. xenopi on bronchoalveolar lavage (BAL) culture. BAL was negative for Mycobacterium tuberculosis (TB) and fungal infection. BAL cytology was also negative for the presence of malignant cells. At that time, her condition was stable and M. xenopi was considered a commensal. Her QuantiFERON-TB Gold test was indeterminate, and tests for serum galactomannan antigen and serum Aspergillus antibody were negative. The legionella urine antigen and HIV test results were also negative.

During current admission, the patient was started on rifampin, isoniazid, ethambutol, and clarithromycin because M. xenopi was clinically significant, causing symptomatic respiratory failure. The patient’s condition improved over the course of the hospital stay, and, subsequently, she was discharged on oral rifampin, isoniazid, ethambutol, and clarithromycin for at least six months of therapy and a possibility of prolongation of treatment based on clinical and radiological improvement.

**Figure 3:**
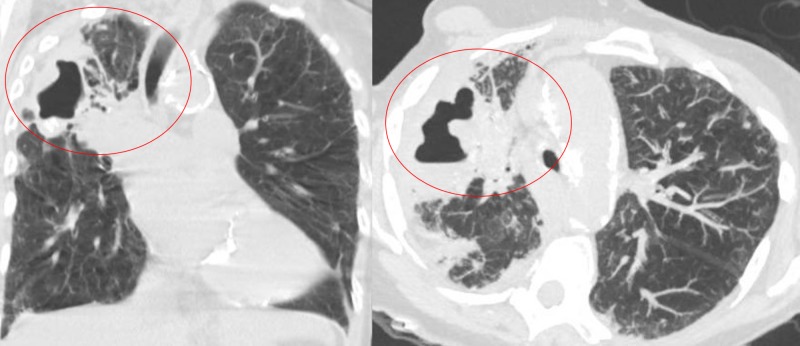
Repeat computed tomography of chest showing right upper lobe consolidation with slight interval enlargement of the right upper lobe cavitary lesion (circled)

One month after discharge, while on treatment, the patient was readmitted for shortness of breath and hypotension, tachypnea, and tachycardia. On a repeat CT scan of her chest, we noted right upper lobe consolidation with slight interval enlargement of the right upper lobe cavitary lesion (Figure 3). The patient was given broad-spectrum antibiotics for possible bacterial superinfection. The patient improved clinically and was discharged. The patient was advised to have close outpatient follow up with the infectious disease and pulmonary teams.

## Discussion

M. xenopi was first identified in 1959 by Schwabacher from lesions on the skin of a South African toad and named after the frog species, Xenopus laevis [5]. M. xenopi is an acid-fast, slow-growing, non-tuberculous bacillus and often considered a commensal, opportunistic or nosocomial pathogen. The usual route of infection for pulmonary diseases is the inhalation of infected aerosol airborne particles. Approximately 50% of isolates are reported to be clinically significant, and most patients have underlying lung disease or other comorbidities [6]. Clinically, M. xenopi infections can present with insidious onset of variable symptoms like a cough, dyspnea, generalized weakness, decreased appetite, weight loss, and hemoptysis. Clinical and radiological pictures in M. xenopi infection are not easily distinguishable from TB or other NTM infection. M. xenopi infection is challenging to diagnose and requires microbiological assessment and multispecialty involvement.

M. xenopi is an uncommon pathogen that most frequently causes pulmonary infection. However, extrapulmonary cases with bone and joint involvement have been reported. It can present with different clinical and radiological patterns. A cavitary form is seen in patients with pre-existing pulmonary disease; a solitary nodular form in immunocompetent patients or an acute diffuse infiltrate form in immunosuppressed patients [7]. However, the most common radiological finding is upper lobe/apical fibro-cavitation [3] whereas MAC is more often seen presenting with nodular bronchiectasis [1].

The treatment success rate in patients with pulmonary M. xenopi ranges from 8.8% to 73% [2] and has high all-cause mortality from 57% [8] to 69.1% [7] in HIV-negative individuals, higher than other NTM. The optimal treatment for M. xenopi has not been established. The ATS has proposed a three-drug regimen with clarithromycin, rifampin, and ethambutol [3]. The British Thoracic Society (BTS) guidelines recommend a four-drug regimen, including a macrolide (azithromycin or clarithromycin), ethambutol, rifampin, and either a fluoroquinolone (moxifloxacin or ciprofloxacin) or isoniazid [9]. Addition of amikacin is suggested in individuals with severe M. xenopi pulmonary disease that have acid-fast bacilli smear sputum samples, lung cavitation, severe symptoms, and signs of systemic illness [9]. Antibiotic treatment should continue for a minimum of 12 months after culture conversion [3,9].

Localized surgical resection of the affected lung represents an essential adjunct to antibiotic therapy [10]. Surgical resection of the lesion may be appropriate in patients who have sufficient lung function and fail to respond to antibiotics [3]. Functional and quality of life aspects, potential risks, and benefits of therapy should be considered in the individual evaluation of treatment. The distinction between colonization and disease may be difficult upon isolation of M. xenopi. A patient-centered approach is essential since M. xenopi is often considered a commensal pathogen. When treatment is needed, multidrug therapy based on appropriate susceptibility testing should be utilized. Novel diagnostic and therapeutic modalities are required to improve the management of these complex infections [11]. Moreover, the optimal duration of therapy must be individualized.

## Conclusions

Our case highlights that M. xenopi should be considered as a cause of cavitary lesion, especially in patients with an underlying lung disease and a rare presentation of M. xenopi causing a cavitary lung disease complicated by sepsis, acute hypoxic respiratory failure, and complicated by superinfection. The detection of NTM pulmonary disease does not necessitate treatment. Treatment should be considered for clinically significant infection with underlying lung disease. Currently, the ATS diagnostic criteria for NTM lung disease applicable for MAC, M. kansasii, and M. abscessus is the best tool for determining the clinical relevance of M. xenopi. We strongly recommend increased physician awareness of these diagnostic criteria and management guidelines by the ATS and BTS.
